# Fatigue Behavior of PBO FRCM Composite Applied to Concrete Substrate

**DOI:** 10.3390/ma13102368

**Published:** 2020-05-21

**Authors:** Angelo Savio Calabrese, Tommaso D’Antino, Pierluigi Colombi, Christian Carloni, Carlo Poggi

**Affiliations:** 1 Department of Architecture, Built Environment and Construction Engineering, Politecnico di Milano, 20133 Milan, Italy; angelosavio.calabrese@polimi.it (A.S.C.); tommaso.dantino@polimi.it (T.D.); carlo.poggi@polimi.it (C.P.); 2 Department of Civil Engineering, Case Western Reserve University, Cleveland, OH 44106, USA; christian.carloni@case.edu

**Keywords:** fatigue, bond, modified beam test, fiber-reinforced cementitious matrix (FRCM)

## Abstract

Several reinforced-concrete (RC) structural elements are subjected to cyclic load, such those employed in highway and railroad bridges and viaducts. The durability of these elements may be reduced as a consequence of fatigue, which mainly affects the steel reinforcement. The use of externally bonded (EB) fiber-reinforced cementitious matrix (FRCM) composites allows the moment capacity to be shared by the internal reinforcement and the EB composite, thus increasing the fatigue life of the strengthened RC member. The effectiveness of EB FRCM composites is related to the composite bond properties. However, limited research is currently available on the effect of fatigue on the bond behavior of FRCM-substrate joints. This study provides first the state of the art on the fatigue behavior of different FRCM composites bonded to a concrete substrate. Then, the fatigue bond behavior of a polyparaphenylene benzo-bisoxazole (PBO) FRCM is experimentally investigated using a modified beam test set-up. The use of this set-up provided information on the effect of fiber-matrix interface shear and normal stresses on the specimen fatigue bond behavior. The results showed that fatigue loading may induce premature debonding at the matrix-fiber interface and that stresses normal to the interface reduce the specimen fatigue life.

## 1. Introduction

A large amount of existing bridges and viaducts in western countries are made of reinforced and pre-stressed concrete. As is well known, these structures are subjected to fatigue loads that affect their load-carrying capacity and durability. Moreover, the increasing traffic volume and weight of vehicles induces an increase of the fatigue loads applied to the structure. The combined effect of cyclic (fatigue) loading and steel reinforcement corrosion often results in fatigue crack initiation and propagation within the steel rebar cross-section, eventually resulting in sudden failure of the reinforcement and therefore of the structural element. Fatigue failure of steel rebars in reinforced-concrete (RC) elements represents an important cause of deterioration of highways, railroad bridges, and viaducts [1].

Several strengthening techniques have been proposed to extend the service life of bridge structures and prevent traffic obstruction, most of them aiming at reducing the stress level in the fatigue-cracked rebars. Classical strengthening solutions include post-tensioning of external cables or near surface mounted steel bars [2]. The introduction of composite materials in the civil engineering field provided innovative solutions to extend the fatigue life of RC structural members. Externally bonded (EB) fiber-reinforced polymers (FRP), consisting of high-strength fiber sheets embedded in epoxy resins or pultruded composite strips bonded to the substrate using epoxy resins, have proven to be an efficient and cost-effective strengthening solution to extend the fatigue life of RC members [3,4]. However, FRP composites present some drawbacks, such as the different physico-chemical properties of organic resin and concrete, which is responsible for the poor thermal and vapor compatibility of FRP with concrete and the poor resistance to relatively high temperatures [5,6]. To overcome these issues, a new technology based on the use of high-strength open-mesh fiber textiles embedded within an inorganic matrix, which are referred to as fiber-reinforced cementitious matrix (FRCM) composites [7], has been introduced recently. Although it is well established that externally bonded FRCM composites are effective in increasing both the flexural [8,9,10,11], shear [12,13,14], and axial strength of RC members [15,16,17,18,19], limited work was carried out to investigate the fatigue behavior of these composites.

Failure of FRCM strengthened members is usually caused by debonding of the composite at the matrix-fiber or at the composite-substrate interface [9]. The FRCM-concrete bond behavior was studied mostly using direct shear test set-ups, where the interface is mainly subjected to shear stress. [20,21,22]. Recently, some studies investigated the effect of the stress component normal to the interface on the FRCM-concrete bond capacity using modified beam test set-ups where the FRCM strips connected the two substrate prisms on one side whereas a cylindrical hinge was positioned on the opposite side [23,24,25,26]. Although bond tests do not exactly reproduce the state of stress of the composite and substrate of a real strengthened member, their result is employed to define the composite effective stress, i.e., the maximum stress that can be applied to the EB composite, by numerous design guidelines (see, e.g., [27,28,29]).

The available literature regarding the bond behavior of FRCM composites focuses on the quasi-static monotonic response, while quite limited research was done to investigate the effect of fatigue loading on the FRCM-substrate and matrix-fiber bond behavior [30]. In the present study, first a thorough review of the currently available studies on the fatigue behavior of RC beams strengthened with FRCM composites is presented, pointing out how different parameters, such as the steel reinforcement ratio, load range, and loading frequency, affect the fatigue life and failure mode of the strengthened member. Then, the preliminary results of an ongoing experimental campaign on modified beam specimens with an externally bonded PBO FRCM strip are discussed to provide an insight on the effect of cyclic (fatigue) load on the FRCM-concrete bond behavior. The use of a modified beam test set-up allowed the effect of fiber-matrix interface shear and normal stresses on the specimen fatigue bond behavior to be investigated. The results showed that the normal stress component increased the bond capacity in quasi-static monotonic tests, whereas it induced damage to the fiber that reduced the specimen fatigue life in fatigue tests. These findings indicate that the normal stress component should be accounted for when a cyclic load is applied to the FRCM strengthened member and that further studies are needed to clarify the effect of the fatigue maximum and minimum applied load on the specimen fatigue life.

## 2. Fatigue Strengthening of RC Beam with FRCM Composites: State of the Art

According to ACI 215 [31], the fatigue limit of plain concrete, which corresponds to 10 million cycles of either compressive, tensile, or flexural loads, is estimated as 55% of its static strength, whereas the stress levels of unembedded steel rebars associated with failure in 2 million cycles are approximately 45%–60% of the corresponding yielding stress. Referring to the internal reinforcement, Eurocode 2 [32] requires that steel bars withstand at least 2 million cycles with a stress range higher than or equal to 150 MPa and a recommended maximum fatigue stress of 60% of the bar characteristic yielding strength. Accordingly, if the fatigue stress exceeds this limit, cracks may occur in the reinforcing bar (rebar) and propagate until sudden failure of the element. Rebar crack propagation, which is accelerated by the simultaneous effect of corrosion and applied loads [33,34], results also in a reduction of the member stiffness and local steel concrete debonding.

Four-point bending experimental tests conducted on 2400 mm long RC beam samples showed that the fatigue life of RC beams is characterized by two main stages [35]. In the initial stage, several new concrete cracks formed in the maximum moment region, which determined a significant reduction of the member flexural stiffness. Increasing the number of load cycles, the crack number and flexural stiffness tended to stabilize and the beam entered the second stage, characterized by the steady propagation and widening of the existing cracks toward the compressive zone and by the occurrence of few new cracks. Between the end of the first and the beginning of the second stage, a fatigue crack initiated in the tension steel rebar at the location of the stress concentration due to bar imperfections (usually at the rib root in deformed rebars [1]) and gradually propagated throughout the whole second stage. The beam eventually failed once the propagation of the fatigue crack in the rebar caused the bar rupture and consequent compressive concrete crushing.

To increase the fatigue life of RC beams, EB FRCM composites can be employed to reduce the stress level in the steel reinforcement. FRCM composites can provide an interesting strength contribution, being applied at the outermost fibers (top or bottom) of the cross-section, which entails for a large moment arm associated with their tensile force contribution [29,36,37]. Therefore, they can lessen the stress range in the steel rebar, which in turn limits the crack growth rate, determining a significant extension of the beam fatigue life. Additionally, FRCM composites counteract the opening of flexural cracks in the concrete, delaying in turn fatigue crack propagation in the steel reinforcement, reducing the rebar exposure to the aggressive environment agents, and thus improving the durability of the steel reinforcement [35].

The following sub-sections provide an overview of the main research studies currently available on the fatigue behavior of FRCM-strengthened RC beams.

### 2.1. Database of Existing Results

**Figure 1:**
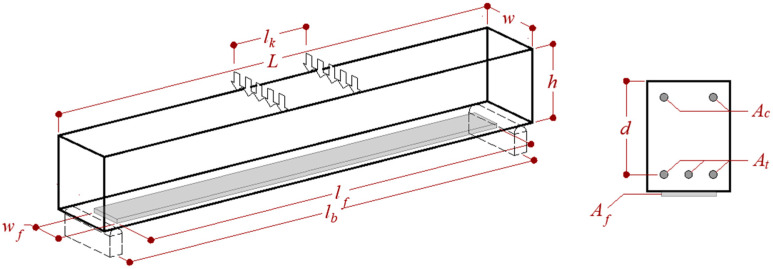
Bending test set-up of the experimental tests collected.

To the best of the authors’ knowledge, five studies that analyzed the effect of various parameters on the fatigue life of FRCM-strengthened RC beams with different geometries subjected to a bending test set-up (see Figure 1) are currently available in the literature [1,35,38,39,40]. The geometrical characteristics of the specimens presented in those five studies are reported in Table 1 with reference to the parameters depicted in Figure 1.

In all tests herein considered, the specimen was subjected to an initial displacement-controlled quasi-static monotonic ramp that was stopped as the mean fatigue load was reached. Then, the specimen was subjected to a sinusoidal cyclic load. The maximum and minimum values of the cyclic load (which define the amplitude) are termed *S_max_* and *S_min_*, respectively. In some studies, the beam was subjected to a long-term sustained load and the steel rebars were also artificially corroded prior to the application of the FRCM composite and subsequent fatigue test.

Table 2 reports the mechanical parameters and results of the specimens considered. For each specimen, the reinforcement ratio *β^f^* is provided [1]:

(1) $$\beta^{f}=\frac{A_{f}E_{f}}{h}\times \frac{d}{A_{t}E_{s}}$$

Equation relates the FRCM and steel reinforcement mechanical ratios, allowing the (potential) FRCM strength contribution with respect to the steel reinforcement one to be expressed.

Specimens with steel bars corroded prior to the fatigue test are indicated with “Y” in the column “Corr”. In Table 2, *P_i_/P_u_* indicates the ratio of the long-term sustained load (when applied) to the ultimate load of the corresponding monotonic test. Finally, *f* and *N_F_* are the cycle frequency and number of cycles at failure (i.e., fatigue life), respectively, whereas the normalized fatigue life $\bar{N}$ is the ratio between the fatigue lives of the FRCM-strengthened and of the corresponding control specimen. When fatigue failure occurred, i.e., the specimen failed for a number of cycles (*N*) less than 2 million, it was due to steel rebar rupture followed by complete debonding of the FRCM at different interfaces. Three different debonding failure modes (FMs) can be identified: (a) at the matrix-fiber interface, (b) at the composite-concrete interface, or (c) at the matrix-fiber interface with damage and eventual rupture of the fiber. It should be noted that local debonding of the FRCM occurred before complete failure of the specimen. The FMs observed are reported in Table 2, where the symbol “>” indicates that the fatigue test was interrupted after 2 million cycles.

### 2.2. Discussion of Existing Results

The fatigue life of the FRCM-strengthened beams analyzed was mainly characterized by three stages [1,35,40]. In the first stage, the occurrence of flexural cracks in the RC beam induced a significant decrease of its flexural stiffness and a consequent increase of the midspan deflection. Simultaneously, the formation of transversal cracks (i.e., orthogonal to the longitudinal fiber yarn direction) in the FRCM matrix was observed. For specimens subjected to long-term sustained load before the application of the fatigue load, the number of new cracks that developed in this first stage was inversely proportional to the amount of sustained load applied [35,38].

The second stage, which covers most of the entire fatigue test, was characterized by a progressive and steady propagation of flexural cracks toward the compression zone. In general, during this stage, a primary flexural crack prevails in the maximum moment region or at the midspan for specimens subjected to a 3-point bending test (*l_k_* = 0 in Figure 1 and Table 1). Elghazy et al. [40] pointed out how the second stage of FRCM-strengthened beams was characterized by a more gradual and steady degradation than that exhibited by unstrengthened beams in the same stage. This effect was attributed to the crack bridging effect of the FRCM composite, which delayed the crack propagation.

During the second stage of FRCM-strengthened beams, fatigue cracks initiated in the steel rebars and steadily propagated with increasing number of cycles, which determined a gradual loss of the specimen flexural stiffness due to the reduction of the bar resisting cross-sectional area. During this stage, the widening of FRCM matrix cracks determined local debonding at the matrix-fiber and composite-concrete interface [1]. In sodium chloride corroded beams, a tendency of developing horizontal cracks in the FRCM reinforcement layer was observed, which resulted in the partial detachment of the FRCM strip from concrete, suggesting a detrimental effect of the chemical agent on the matrix-concrete bond capacity [35].

In the third (final) stage, the rebar fatigue crack growth rate rapidly increased and the collapse suddenly occurred due to the rebar rupture. Simultaneously, the large beam deflection induced complete FRCM debonding according to the failure modes described in the previous section (see Table 2).

**Figure 2:**
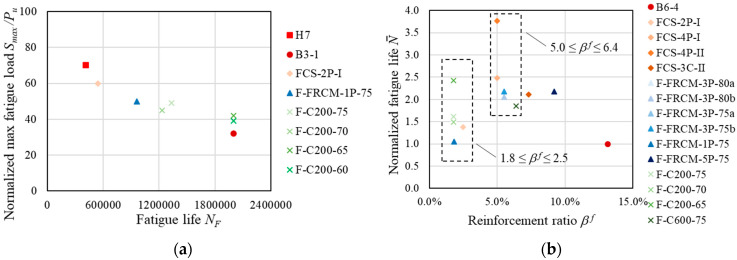
State of the art analysis: (**a**) relationship between normalized maximum applied fatigue load and fatigue life; (**b**) relation between normalized fatigue life and reinforcement ratio.

Figure 2a shows the relationship between the fatigue life, *N_F_*, and the normalized maximum applied fatigue load, *S_max_/P_u_*, of specimens characterized by reinforcement ratios, *β^f^*, between 1.8% and 3.3% and that were not subjected to long-term sustained load before the fatigue test. Figure 2a shows that high *S_max_/P_u_* values determined a significant reduction of the beam fatigue life with respect to that of specimens subjected to low values of *S_max_/P_u_*. Specimens B3-1, F-C200-60, and F-C200-65 did not fail after the target number of cycles, i.e., 2 million cycles, was attained. This shows that *S_max_/P_u_* lower than or approximately equal to 40% did not lead to fatigue failure within 2M cycles, which can be attributed to a stress level in the steel reinforcement lower than the corresponding fatigue limit or endurance.

Figure 2b shows the relationship between the reinforcement ratio, *β^f^*, and the normalized fatigue life, $\bar{N}$, of specimens subjected to a maximum fatigue load, *S_max_*, in the range 40%–60% of the corresponding quasi-static monotonic ultimate load, *P_u_*. Specimens showing a premature failure ($\bar{N}$ < 1, [1]) were not included in Figure 2b. Although the results reported showed a certain scatter (typically observed in fatigue tests), comparison of $\bar{N}$ between specimens characterized by low reinforcement ratios (1.8 ≤ *β^f^* ≤ 2.5) with that of moderately reinforced specimens (5.0 ≤ *β^f^* ≤ 6.4) suggests a general increase of the normalized fatigue life, $\bar{N}$, with the increase of the reinforcement ratio, *β^f^*. However, specimens with a reinforcement ratio *β^f^* > 6.4 did not show an increase of $\bar{N}$ with respect to specimens with 5.0 ≤ *β^f^* ≤ 6.4 (Figure 2b). This result could be attributed to the occurrence of FRCM-concrete debonding that initiated at cracked cross-sections of specimens with a high reinforcement ratio. Indeed, experimental results showed that increasing the number of fiber layers does not lead to a proportional increase of the FRCM capacity, since the failure mode may vary and premature debonding may occur [9]. As pointed out in [40], the capacity of the FRCM composite of reducing the stress in the steel reinforcement is affected by the composite-concrete stress-transfer characteristics, which mainly depend on the matrix-fiber impregnation capability [42,43] and on the matrix compatibility with the substrate [35].

The results analyzed suggest a relationship between the reinforcement ratio and the FRCM failure mode. High *β^f^* led to debonding at the composite-concrete interface (failure mode b in Table 2), due to the high FRCM stiffness [9,21], whereas low *β^f^* determined matrix-fiber debonding (failure mode a in Table 2). However, it should be noted that complete FRCM debonding never occurred before steel rebar fatigue failure.

## 3. Experimental Program

Based on the results discussed in the previous section, the effectiveness of FRCM strengthening application appears strongly related to its bond properties. Therefore, study of the fatigue bond behavior of FRCM composites may provide important information to capture correctly the FRCM contribution to the fatigue life of FRCM-strengthened RC beams. In this section, some preliminary results of an ongoing experimental campaign on PBO FRCM-concrete joints are presented and discussed. In particular, six modified beam tests were performed, three subjected to quasi-static monotonic loading and three to fatigue loading. The average ultimate load of the quasi-static monotonic tests was used as a reference to define the parameters of the fatigue load range applied to the specimens subjected to fatigue loading. Specimens were named according to the notation MB, X, Y, B, F, n, where MB (=modified beam) indicates the test setup, X is the bonded length (in mm), Y is the bonded width (in mm), B (=bare fibers) indicates the strip layout (see next section), F (if present) indicates a fatigue test, and n is the specimen number.

### Specimen Geometry and Materials

**Figure 3:**
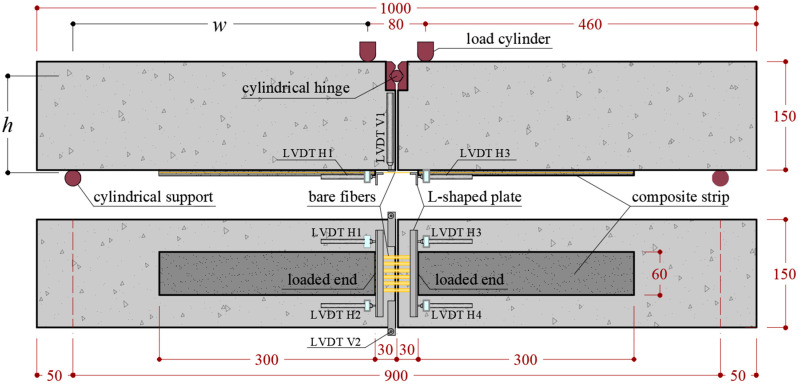
Modified beam test geometry (dimensions in mm).

Each specimen (see Figure 3) consisted of two concrete prisms that formed a beam as they were joined by a cylindrical hinge (at the midspan of the beam) placed near the compression side and by a FRCM strip applied at the bottom (tension) side. The midspan concrete discontinuity in the modified beam simulates the presence and opening of a flexural crack. Employing two separate prisms connected by the cylindrical hinge and by the composite strip, the position of the compression and tensile stress resultants at the midspan cross-section is always known, which allows the uncertainties associated with the definition of the stress resultant in the compressed concrete in continuous or notched beam tests to be eliminated [29,44]. This test was originally proposed to study the bond behavior of steel bars embedded in concrete [45] and was subsequently extended to the study of externally bonded [46] and near surface mounted [47] FRP composites. Recently, it was adopted to study the bond behavior of FRCM composites applied to concrete and masonry substrates to investigate the effect of interface normal stresses on the specimen capacity [24,25].

In this paper, the concrete prisms had nominal dimensions equal to 150 × 150 (cross-section) × 500 (length) mm^3^. The concrete average compressive strength was measured on six 150 mm side cubes cast from the same batch of concrete used to cast the prisms and was equal to 37.9 MPa (coefficient of variation, CoV, equal to 6.02%). The FRCM composite was comprised of an open-mesh unbalanced PBO textile embedded within two layers of cement-based mortar. The PBO textile had a regular rectangular mesh with a clear space between bundles of 5 mm in the main (longitudinal) directions and 15 mm in the transverse direction. The area of each longitudinal bundle was 0.46 mm^2^ and each specimen strip included 6 longitudinal yarns, resulting in an overall fiber cross-sectional area *A_f_* = 2.76 mm^2^. The composite overall bonded length and width were 600 mm (300 mm on each prism) and 60 mm, respectively. Close to the beam midspan, a 60 mm long portion of textile was not embedded within the matrix, i.e., the fibers were left bare [24]. This discontinuity in the matrix allowed for a direct measurement of the relative slippage between fibers and matrix at the beginning of the bonded region of each prism (termed loaded end in Figure 3).

The bare PBO fiber average tensile strength and elastic modulus, measured using tensile testing of textile strips with different widths, were equal to 3015 MPa (CoV = 6.8%) and 206 GPa (CoV = 6.5%), respectively [22]. The matrix flexural and splitting strength were 28.5 MPa and 3.5 MPa, respectively [48]. The tensile behavior of the FRCM composite was previously studied by tensile testing of FRCM coupons using both the clamping-grip and the clevis-grip methods provided by the Italian initial type testing procedure [49] and U.S. acceptance criteria [50] for FRCM composites, respectively. Clamping-grip tests showed a trilinear stress-strain response with failure due to rupture of the embedded fiber textile [41], while clevis-grip tests showed a bilinear behavior with failure due to debonding at the matrix-fiber interface [41,51]. Further details of these test results can be found in [41,51].

A 4-point bending set-up was used. The beams were placed on two cylindrical supports 900 mm apart, whereas the load was applied by two steel cylinders connected to the machine by a spherical hinge. Six linear variable displacement transformers (LVDTs), named H1, H2, H3, H4, V1, and V2 (Figure 3), were used to measure the displacement of the fiber textile with respect to the concrete support and the midspan vertical deflection of the specimen. Two LVDTs were attached to each concrete block at the strip loaded ends (H1 and H2 on one side and H3 and H4 on the opposite side, see Figure 3) and reacted off of L-shaped steel plates bonded to the midspan bare fibers at the FRCM loaded ends. The remaining two LVDTs (V1 and V2) were used to measure the midspan vertical deflection on each side of the specimen.

## 4. Quasi-Static Monotonic Tests

**Figure 4:**
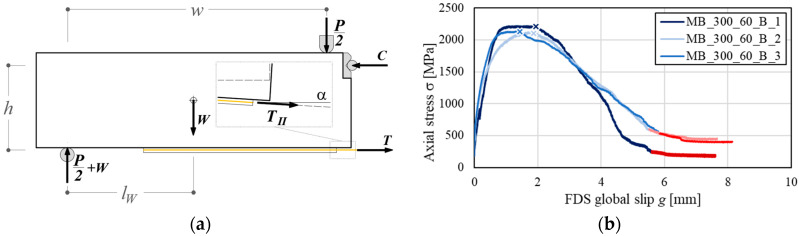
Modified beam tests: (**a**) free body diagram; (**b**) σ-*g* responses at fully debonded side (FDS) of quasi-static monotonic tests (the peak stresses σ* are indicated with a “x” marker whereas the curve portions characterized by first derivative values within the range (−200,0) are depicted in red).

Quasi-static monotonic tests were first conducted to obtain the specimen load carrying capacity. Tests were performed by monotonically increasing the vertical displacement (stroke) of the machine head at a constant rate of 0.2 mm/min. The fiber axial force parallel to the strip direction at the loaded end, *T*_II_, associated with the load *P* applied by the testing machine, was computed by enforcing the equilibrium of the free-body diagram of Figure 4a:

(2) $$T_{Ⅱ}=T\cos\alpha =\frac{P\times w+2W\times l_{W}}{2h}$$

where *α* is the specimen deflection angle [24], *W* is the block self-weight, *l_W_* is the horizontal distance of the center of gravity of the block to the closest support axis, *w* is the distance of the line of action of the load to the axis of the closest cylindrical support, and *h* is the vertical distance of the axis of the hinge to the fiber plane.

All specimens failed due to debonding of the fiber from the embedding matrix, as observed in single- or double-lap direct shear tests of the same FRCM composite including one or two layers of fiber [22]. The fiber stress σ-global slip *g* responses of the three monotonic tests are reported in Figure 4b, where σ is the ratio between *T*_II_ and the total fiber area *A_f_* and *g* is the average displacement of the two LVDTs (either H1 and H2 or H3 and H4) located at the loaded end of the strip that exhibited complete debonding of the fibers at the end of the test. The specimen side where full debonding occurred is named fully debonded side (FDS) [24]. All specimens showed an initial linear branch (Figure 4b) associated with the elastic behavior of the matrix-fiber interface. Referring to this branch, the averages of the displacements measured using either LVDTs H1 and H2 or LVDTs H3 and H4 were almost identical, which indicates symmetric slippage of the fibers at the two sides of the specimen. With increasing the machine stroke, micro-cracking at the matrix-fiber interface occurred and the load response became non-linear. In this branch, the global slip at the FDS started increasing at a higher rate with respect to that on the opposite side, named partially debonded side (PDS) [24]. As the machine stroke increased, the specimen attained the peak stress σ*. Further increase of the machine stroke after σ* determined a reduction of the applied load with increasing global slip at the FDS, whereas the slip at the loaded end of the PDS (not shown in Figure 4b) remained approximately constant or slightly decreased, probably due to the recovery of the elastic deformation of the fibers. The applied stress eventually plateaued at a value σ*_f_* associated with the presence of friction at the matrix-fiber interface [23,52] on the FDS. For specimen MB_300_60_B_3, the applied friction stress at the completion of the test was lower than that of specimens MB_300_60_B_1 and 2 (Figure 4b). This difference was attributed to the rupture of some fiber filaments caused by the presence of a stress component normal to the fiber textile plane that arose at the loaded end as a consequence of the relative rotation of the prisms [24].

The peak and friction stresses of all quasi-static monotonic tests are reported in Table 3 together with their average values for the three specimens and corresponding coefficient of variation (CoV). Following the procedure proposed in [23,52,53], σ*_f_* was computed as the average of the applied stress within the range of *g* where the first derivative of σ(*g*) with respect to *g* was within (−200,0). This range is highlighted in Figure 4b at the tail of each curve.

The peak stress values obtained by the modified beam tests were higher than those of direct-shear tests of FRCM-concrete joints with the same PBO FRCM composite available in the literature. If the average peak stress reported in Calabrese et al. (2020) [54] for PBO FRCM-concrete joints with a bonded length of 300 mm, i.e., 1858 MPa, is considered, the increase of bond capacity obtained with the modified beam tests is approximately 16%. This increase can be attributed to the presence of the normal stress component at the matrix-fiber interface, induced by the relative rotation of the prisms.

## 5. Fatigue Tests

**Figure 5:**
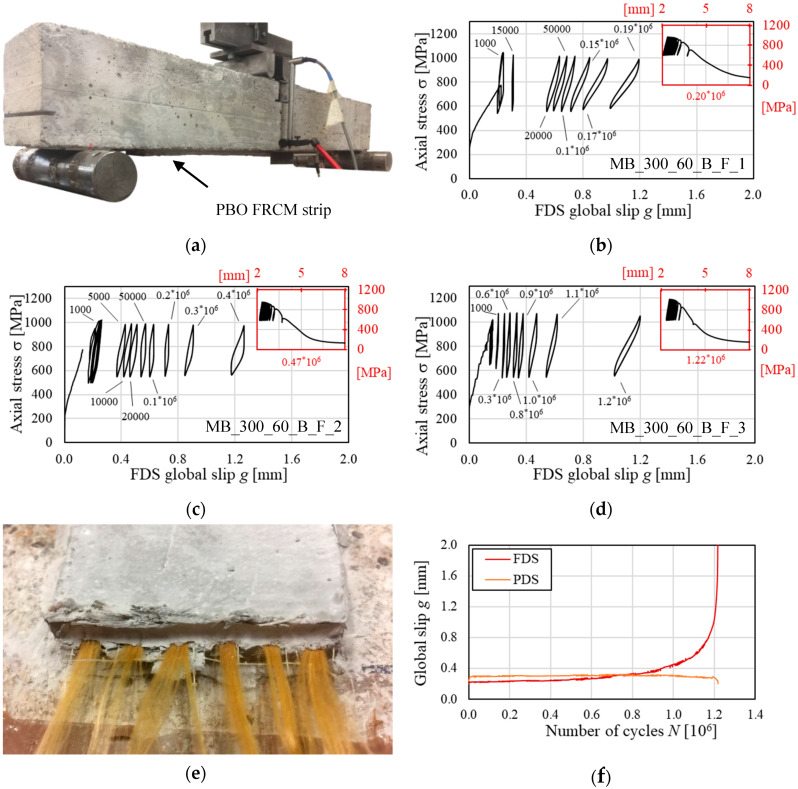
(**a**) Photo of specimen MB_300_60_B_F_3 before the beginning of the test. Load responses of specimen (**b**) MB_300_60_B_F_1, (**c**) MB_300_60_B_F_2, and (**d**) MB_300_60_B_F_3. (**e**) Failure of PBO fibers and (**f**) global slip vs. number of cycles for specimen MB_300_60_B_F_3.

Specimens MB_300_60_B_F_1, 2, and 3 were subjected to a fatigue test. A photo of specimen MB_300_60_B_F_3 before the beginning of the test is shown in Figure 5a. The main geometrical properties and the number of cycles at failure, *N_F_*, of each specimen are reported in Table 4.

The specimens were initially loaded by monotonically increasing the machine stroke at a rate of 0.2 mm/min until the mean fatigue load was attained. Then, a sinusoidal load was applied until 2 × 10^6^ cycles or specimen failure were reached. The minimum and maximum applied load in the fatigue cycles were equal to 25% and 50% of the average peak load of the quasi-static monotonic tests, respectively. The fatigue load range was determined considering an FRCM-strengthened member for which the composite provides a (relatively) small contribution to the load-carrying capacity under dead loads and is stressed up to half of its quasi-static monotonic load-carrying capacity, σ*, under service loads. The fatigue load range determined corresponded to an axial stress in the fibers ranging between σ*_min_*= 550 MPa and σ*_max_*= 1050 MPa. Furthermore, based on load frequencies generally observed in civil engineering structures, which were reported to vary between 1 and 5 Hz [30], a loading frequency equal to 3 Hz was adopted.

The three specimens subjected to the fatigue test failed due to matrix-fiber debonding and eventual rupture of the fibers at the strip loaded ends (failure mode c in Section 2.1). Figure 5b–d show the load responses of the specimens subjected to fatigue cycles in terms of axial stress, σ, in the fibers versus global slip, *g*, on FDS. In particular, Figure 5b–d feature the following portion of the response: (i) the load response associated with the initial quasi-static monotonic test, (ii) representative 10-cycle blocks selected at different number of cycles, and (iii) the σ-*g* response of the 10 cycles preceding failure of the specimen. During the first part of the fatigue test, the behavior of the FRCM strips attached to the two concrete prisms was symmetrical, as it could be inferred from the readings of LVDTs H1 and H2 and LVDTs H3 and H4. As the number of load cycles increased, the global slip measured at one side of the specimen, the FDS, started to increase at a higher rate with respect to that measured on the opposite side (i.e., the PDS) and the specimens entered in the second part of the test. The global slip increase on the FDS could be attributed both to a progressive debonding at the matrix-fiber interface and to damage and eventual rupture of some fiber filaments due to the interlocking action with the embedding matrix [20,30]. Due to the progressive debonding and fiber damage, the stiffness of the specimen decreased, as can be inferred by the decreasing slope of the 10-cycle blocks as the global slip increased. The decrease in stiffness was associated with an increase of area enclosed by each cycle in Figure 5b–d. Further analyses are needed to clarify whether the progressive matrix-fiber debonding or damage and rupture of the fiber filaments was mainly responsible for the progressive degradation of the specimen mechanical properties during the fatigue steady stage. With the increase of the number of cycles, the specimens entered a non-steady stage where *g* rapidly increased on the FDS until sudden collapse due to complete rupture of all fiber filaments, as shown in Figure 5e for specimen MB_300_60_B_F_3, occurred. Since the fatigue test was load-controlled, no residual constant applied stress could be observed at the end of the test. In fact, due to the progressive fiber filaments rupture, the specimens were no longer able to sustain the stress within the fatigue range applied, which determined the complete failure of the fibers.

The global slip, *g*, computed as the average of the displacements of the two LVDTs on each specimen side (i.e., FDS and PDS) is depicted with respect to the number of cycles, *N*, for specimen MB_300_60_B_F_3 in Figure 5f. The global slip on the FDS was consistent with the global slip on the PDS up to approximately 0.55 × 10^6^ cycles, which indicates that the two strips on each side of the specimen behaved symmetrically during the first part of the test. As the number of cycles increased, *g* started to increase on the FDS, whereas it remained approximately constant on the PDS. When it exceeded 0.90 × 10^6^, the specimen entered the non-steady state, where the crack growth rate on the FDS that can be linked to d*g*/d*N* increased rapidly until failure of the specimen.

**Figure 6:**
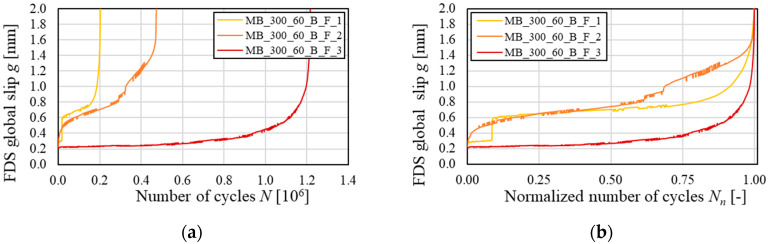
FDS global slip vs. (**a**) number of cycles and (**b**) normalized number of cycles for specimen subjected to fatigue test.

Although the same failure mode occurred for all specimens, the number of cycles at failure, *N_F_*, was different (see Table 4). Figure 6a shows the global slip on the FDS versus the number of cycles for all specimens subjected to fatigue, whereas Figure 6b shows the same FDS global slip with respect to the normalized number of cycles *N_n_* = *N/N_F_*. Specimen MB_300_60_B_F_1 failed after the lowest number of cycles, i.e. *N_F_* = 0.20 × 10^6^ (Table 4). Specimens MB_300_60_B_F_2 and 3 failed at *N_F_* equal to 0.47 × 10^6^ and 1.22 × 10^6^, respectively, which are still limited compared to the target fatigue life of 2 × 10^6^ cycles. In general, specimens that experienced high values of FDS global slip at a low number of cycles provided a short fatigue life. This might indicate that the specimen degradation occurred progressively and not as a consequence of sudden damage. In specimen MB_300_60_B_F_1, an instantaneous increase of the FDS global slip from 0.3 mm to 0.6 mm was observed at the beginning of the test due to the opening of a matrix crack along the textile plane at the loaded end. This type of crack, which was not observed in other specimens subjected to either fatigue or quasi-static monotonic loading, was attributed to poor adhesion between the internal, i.e., in contact with the substrate, and external matrix layer. However, the presence of the crack affected the response of the specimen, which showed a FDS *g*-*N* response during the steady stage with a slope similar to that of specimen MB_300_60_B_F_2 and significantly higher than that of specimen MB_300_60_B_F_3 (see Figure 6a). The steady stage ended when the FDS global slip attained approximately 0.15, 0.45, and 0.80 mm for specimens MB_300_60_B_F_1, _2, and _3, respectively.

The scatter between the results obtained did not allow a clear effect of the fatigue load on the FRCM-concrete bond behavior to be identified. Comparisons of these results with those of direct shear tests on the same PBO FRCM composite [30] will help the effect of the test set-up and FRCM strip layout on the fatigue behavior of FRCM-concrete joints to be understood. Further investigations are needed to clarify the maximum load range that can be applied to the externally bonded FRCM strip to guarantee an adequate fatigue life of the RC strengthened member.

## 6. Conclusions

In this study, an overview of the effect of different parameters on the bond between FRCM composites and RC beams collected from the literature was presented. Furthermore, the preliminary results of an experimental campaign to study the fatigue response of modified beam test specimens comprising PBO FRCM strips and concrete prisms were discussed. Based on the literature review and results herein presented, the following conclusions can be drawn:

- The fiber reinforcement ratio *β^f^* has an important role in the fatigue life of FRCM-strengthened RC beams due to its influence on the composite (debonding) failure mode. As a consequence, high reinforcement ratios did not provide significant increase in the specimen fatigue life due to the attainment of the composite bond capacity.
- The quasi-static monotonic modified beam tests failed due to debonding at the matrix-fiber interface and, in one case, rupture of the fiber filaments, which was attributed to the presence of a stress component normal to the fiber textile plane that damaged the fiber filaments.
- The fatigue modified beam tests failed due to rupture of the fiber filaments with a number of cycles at failure between 0.20 × 10^6^ cycles and 1.22 × 10^6^ cycles. The failure mode did not allow verification of whether the progressive matrix-fiber debonding or damage of the fiber was mainly responsible for the progressive degradation of the specimen stiffness.

The results obtained are valid on for the PBO FRCM composite studied. The mode of failure observed, i.e., debonding at the matrix-fiber interface, showed the importance of the matrix-fiber bond properties and indicates that new experimental tests are needed if the fiber or matrix are varied.

## Figures and Tables

**Table 1:** Geometrical parameters depicted in Figure 1.

Reference	Set-Up		RC Beam						FRCM	
	*l_b_* (mm)	*l_k_* (mm)	*L* (mm)	*w* (mm)	*H* (mm)	*D* (mm)	*A_c_* (mm^2^)	*A_t_* (mm^2^)	*l_f_* (mm)	*w_f_* (mm)
[35]	2200	800	2400	120	230	205	101	308	2000	120
[39]	2032	254	2133	203	305	268	157	236	1880	203
[40]	2560	800	2800	2345	250	225	101	402	2400	150
[1]	1524	0	1829	2972	305	268	142	213	1524	152
[38]	1524	0	1829	2345	305	268	142	213	1524	152

**Table 2:** Main parameters and results of the experimental tests compiled.

Ref.	Specimen	FRCM	*E_f_* (GPa)	*A_f_* (mm^2^)	*β^f^* (%)	*Corr*	*P_i_/P_u_*	*S_min_/P_u_* (%)	*S_max_/P_u_* (%)	*F* (Hz)	*N_F_* (10^4^)	$\bar{N}$ (%)	FM
[35]	H2	control	-	-	-	Y	0.2	20	70	3	27.9	-	-
	H3	C	80 ^‡^	9.00	2.9	Y	0.2	20	70	3	33.7	1.25	a + c
	H4	C	80 ^‡^	9.00	2.9	Y	0.4	20	70	3	88.4	3.28	a + c
	H5	C	80 ^‡^	9.00	2.9	Y	0.6	20	70	3	60.0	2.22	a + c
	H6	control	-	-	-	-	-	20	70	3	31.5	-	-
	H7	C	80 ^‡^	9.00	2.9	-	-	20	70	3	41.5	1.32	a + c
[39]	B1-0	control	-	-	-	-	-	16	32	5	200.0	-	-
	B3-1	PBO	127	11.28	3.3	-	-	16	32	5	200.0	1.00	>
	B4-1	PBO	127	11.28	3.3	Y	-	16	32	5	200.0	1.00	>
	B5-1	PBO	127	11.28	3.3	Y	0.4	16	32	5	200.0	1.00	>
	B6-4	PBO	127	45.12	3.3	-	-	20	40	5	200.0	1.00	>
	B7-4	PBO	127	45.12	3.3	Y	-	20	40	5	200.0	1.00	>
	B8-4	PBO	127	45.12	3.3	Y	0.4	20	40	5	200.0	1.00	>
[40]	FCU	control	-	-	-	-	-	21	60	2	39.6	-	-
	FCS-2P-I	PBO	121	15.00	2.5	Y	-	21	60	2	54.5	1.38	b
	FCS-4P-I	PBO	121	30.00	5.0	Y	-	21	60	2	98.4	2.49	b
	FCS-4P-II	PBO	121	30.00	5.0	Y	-	21	60	2	149.3	3.77	b
	FCS-3C-II	C	75	70.65	7.3	Y	-	21	60	2	83.4	2.11	b
[1]	F-CON-0-75a	control	-	-	-	-	-	13	48	2	91.9	-	-
	F-FRCM-3P-90	PBO	128	20.98	5.5	-	-	11	49	2	49.2	0.54	b
	F-FRCM-3P-85	PBO	128	20.98	5.5	-	-	11	46	2	56.2	0.61	b
	F-FRCM-3P-80a	PBO	128	20.98	5.5	-	-	11	44	2	200.0	2.18	>
	F-FRCM-3P-80b	PBO	128	20.98	5.5	-	-	11	44	2	189.0	2.06	b
	F-FRCM-3P-75a	PBO	128	20.98	5.5	-	-	11	41	2	200.0	2.18	>
	F-FRCM-3P-75b	PBO	128	20.98	5.5	-	-	11	41	2	200.0	2.18	>
	F-FRCM-1P-75	PBO	128	7.00	1.8	-	-	12	50	2	96.2	1.05	a
	F-FRCM-5P-75	PBO	128	34.96	9.2	-	-	15	46	2	200.0	2.18	>
[38]	F-CON-75	control	-	-	-	-	-	14	51	2	82.4	-	-
	F-C200-75	C	65	13.38	1.8	-	-	13	49	2	133.4	1.62	a
	F-C200-70	C	65	13.38	1.8	-	-	13	45	2	123.1	1.49	a
	F-C200-65	C	65	13.38	1.8	-	-	13	42	2	200.0	2.43	>
	F-C200-60	C	65	13.38	1.8	-	-	13	39	2	200.0	2.43	>
	F-C600-75	C	64	47.73	6.4	-	-	11	41	2	152.6	1.85	b
	F-C600-70	C	64	47.73	6.4	-	-	11	38	2	195.9	2.38	b
	F-C600-65	C	64	47.73	6.4	-	-	11	35	2	200.0	2.43	>

Note: C = carbon, PBO = polyparaphenylene benzo-bisoxazole, control = non-strengthened beam; *E_f_* = FRCM cracked elastic modulus according to ACI 549.4R-13 (2013); *A_f_* = fiber cross-sectional area; *β^f^* = reinforcement ratio according to Equation (1); ^‡^ not declared in the original publication and assumed equal to that obtained for carbon FRCM in [41].

**Table 3:** Results of quasi-static monotonic modified beam tests.

Specimen	Bonded Length ^†^ (mm)	Bonded Width (mm)	σ* (MPa)	σ*_f_* (MPa)
MB_300_60_B_1	300	60	2220	199
MB_300_60_B_2	300	60	2124	481
MB_300_60_B_3	300	60	2135	430
Average			2160	370
CoV (%)			2.42	40.6

**^†^** Bonded length of each side of the specimen (Figure 3).

**Table 4:** Results of fatigue modified beam tests.

Specimen	Bonded Length ^†^ (mm)	Bonded Width (mm)	σ*_min_* (MPa)	σ*_max_* (MPa)	*N_F_* (10^6^)
MB_300_60_B_F_1	300	60	550	1050	0.20
MB_300_60_B_F_2	300	60	550	1050	0.47
MB_300_60_B_F_3	300	60	550	1050	1.22

^†^ Bonded length of each side of the specimen (Figure 3).
